# Epidemiology and prediction of non-targeted bacteria by the filmarray pneumonia plus panel in culture-positive ventilator-associated pneumonia: a retrospective multicentre analysis

**DOI:** 10.1186/s13613-025-01468-6

**Published:** 2025-04-28

**Authors:** F. Berteau, A. Kouatchet, Y. Le Gall, C. Pouplet, A. Delbove, C. Darreau, J. Lemarie, F. Jarousseau, F. Reizine, C. Giacardi, G. Allo, C. Aubron, M. Eveillard, V. Dubee, R. Mahieu

**Affiliations:** 1 Réanimation Polyvalente et Soins Continus, Centre Hospitalier des Pays de Morlaix, 15 Rue de Kersaint- Gilly, Morlaix, 29600 France; 2 Médecine Intensive– Réanimation et Médecine Hyperbare, Centre Hospitalier Universitaire d’Angers, 4 rue Larrey, Angers, 49933 France; 3 Réanimation-Unité de Soins Continus, Centre Hospitalier de Bretagne Sud, 5 Avenue de Choiseul, Lorient, 56000 France; 4 Réanimation Polyvalente, Centre Hospitalier Départemental Vendée, Boulevard Stéphane Moreau, La Roche Sur Yon, 85925 France; 5 Réanimation Polyvalente, Centre Hospitalier Bretagne Atlantique, 20 Boulevard Général Maurice Guillaudot, Vannes, 56017 France; 6 Réanimation Médico-Chirurgicale, Centre Hospitalier du Mans, 194 Avenue Rubillard, Le Mans, 72037 France; 7 Médecine Intensive Réanimation, Centre Hospitalier Universitaire de Nantes, 30 Boulevard Jean Monnet, Nantes, 44000 France; 8 Réanimation Unité de Surveillance Continue, Centre Hospitalier de Cholet, 1 Rue Marengo, Cholet, 49325 France; 9 Service des Maladies Infectieuses et Réanimation Médicale, Centre Hospitalier Universitaire de Rennes, 2 Rue Henri le Guilloux, Rennes, 35033 France; 10 Hôpital d’Instruction des Armées Clermont-Tonnerre, Rue du Colonel Fonferrier, Réanimation, Brest, 29240 France; 11 Réanimation– Unité de Soins Continus, Centre Hospitalier de Saint Malo, Rue de la Marne, Saint Malo, 35400 France; 12 Médecine Intensive Réanimation, Boulevard Tanguy Prigent, Centre Hospitalier Universitaire de Brest, Université de Bretagne Occidentale, Brest, 29609 France; 13 Laboratoire de Biologie des Agents Infectieux, Unité Bactériologie, Centre Hospitalier Universitaire d’Angers, 4 rue Larrey, Angers, 49933 France; 14 Service de Maladies Infectieuses et Tropicales, Centre Hospitalier Universitaire d’Angers, 4 rue Larrey, Angers, 49933 France

**Keywords:** Ventilator-associated pneumonia, Multiplex polymerase chain reaction, Bacteria, Intensive care units, Antibiotic therapy, Rapid diagnosis

## Abstract

**Background:**

Ventilator-associated pneumonia (VAP) is a prevalent nosocomial infection in intensive care units (ICUs) with significant impacts on patient outcomes and healthcare costs. Multiplex PCR could allow for personalized empirical treatment of VAP and optimize antibiotic therapy.

**Methods:**

This multicenter retrospective study analyzed culture-positive VAP cases from January 2016 to March 2021 across 12 ICUs in France. The prevalence of non-targeted bacteria was evaluated according to the bacterial species included in the BioFire^®^ FilmArray^®^ Pneumonia Panel (FAPPP), and associated risk factors were identified. A non-targeted bacteria was defined as a bacterial species isolated during VAP, not included in the FilmArray panel, but considered by the clinician in the final antibiotic therapy.

**Results:**

Among 332 patients with 385 culture-positive VAP episodes, non-targeted pathogens were observed in 23% of cases (87/385) and represented 21% (110/534) of isolated bacteria (After excluding bacteria with low pathogenicity, the rate of VAP with a non-targeted bacterium was 21%). The most common non-targeted bacteria identified were *Stenotrophomonas maltophilia* (22%), *Citrobacter koseri*, and *Hafnia alvei*. Gram stain results poorly correlated with definitive cultures (42% of concordance). The proportion of culture-positive VAP with non-targeted bacteria varied significantly between ICUs, ranging from 12 to 37%, (*p* = 0.013). Polymicrobial culture-positive VAP had a twofold higher risk of non-targeted bacteria (47% vs. 25%, *p* < 0.001). In the multivariate analysis, in-ICU antibiotic exposure was associated with a twofold increased risk of non-targeted bacteria (25.3% vs. 12.9%, *p* = 0.042), and age over 70 years was associated with a threefold increased risk (*p* = 0.027). Among the 48 culture-positive VAP cases with ineffective empiric treatment, *Pseudomonas aeruginosa* (22%), *Stenotrophomonas maltophilia* (14%), and *Enterobacter cloacae complex* (8%) were the most frequent bacteria. Additionally, 67% of the culture-positive VAP cases with ineffective empirical antibiotic therapy involved targeted bacteria, of which 59% could have received effective empirical antibiotic therapy if panel results had been available, according to bacterial species identification and current guidelines.

**Conclusions:**

A significant rate of culture-positive VAP cases with non-targeted bacteria was observed in this study, raising concerns about the interpretation of FAPPP results. Only positive FAPPP results may assist clinicians in the early personalization of antibiotic therapy for VAP.

**Supplementary Information:**

The online version contains supplementary material available at 10.1186/s13613-025-01468-6.

## Introduction

Ventilator-associated pneumonia (VAP) is the most common nosocomial infection in intensive care units (ICU). The incidence of VAP exceeds 18 per 1000 days of MV in Europe [1]. This infection has an estimated attributable mortality of 5–13% [2, 3]. VAP is associated with increased morbidity, extended duration of MV, and prolonged stays in both the ICU and hospital [1, 4]. It also encourages increased antibiotic use in the intensive care unit, resulting in an overall increase in hospitalization costs [5].

Diagnosing VAP can be challenging [6], and a microbiological result of deep respiratory sampling is often required to confirm diagnosis. Their results help to adjust empirical, usually broad-spectrum, antibiotic therapy in this nosocomial setting.

It is currently recommended to start antibiotic therapy as soon as the respiratory sample is collected. The choice of this empirical antibiotic therapy is based on certain patient risk factors such as comorbidity and their severity, risk factors for MDR pathogens, and the local pattern of antimicrobial susceptibility. Empirical antibiotic therapy is generally based on a non-pseudomonal third generation cephalosporin or broader antibiotic therapy for critically ill patients or those at risk of MDR pathogens [7, 8].

Optimizing the administration of empirical antibiotic therapy can potentially mitigate the complications associated with ventilator-associated pneumonia (VAP) [9]. Multiplex PCR (mPCRs) panels (performed on deep respiratory samples) include dozen bacterial species targets and some resistance genes. The selected targets are based on the aggregated epidemiological data of VAP and show excellent performance compared to standard culture techniques [10]. mPCR techniques provide immediate information (within 1 to 4 h) after sampling, allowing for the consideration of personalized empirical antibiotic therapy. For decades, only epidemiological data and Gram staining results have guided the choice of empirical antibiotic therapy. Species-level identification of bacteria potentially involved in VAP would enable better adjustment of empirical therapy. One limitation of these mPCR techniques is their lack of comprehensiveness, raising concerns about misinterpreting the absence of bacteria in the case of a negative result.

The FilmArray^®^ Pneumonia Panel Plus^®^ (FAPPP) has 15 targets for bacteria involved in VAP and detects 7 resistance genes (Supplementary, Table S1). The test provides semi-quantitative results for these 15 bacteria to help distinguish infection from colonization (but no threshold has been validated). However, some bacteria may be absent from these panels (non-targeted bacteria), leading to a risk of false negative results. The variations in VAP epidemiology could cause differences in the performance of these mPCRs.

We aimed to compare the rate of culture-positive VAP with at least one non-targeted pathogen identified by the FAPPP across different ICUs in a large region of western France. Additionally, we identified the main bacteria responsible for these culture-positive VAP cases and the factors associated with non-targeted pathogens.

## Methods

We conducted a multicenter retrospective study of bacterial VAP from January 1, 2016, to March 31, 2021. Seventeen medical French and/or polyvalent ICUs in the Pays de la Loire and Brittany regions (with a population of more than 7 million people) were eligible. Culture-positive VAP cases were identified by analysis of ICU patient medical records and respiratory samples from bacteriological laboratories.

The inclusion criteria were defined as follows: (i) Any patient meeting the diagnostic criteria for VAP ([6, 11]) and (ii) Identification of at least one bacterial species in a respiratory sample at a significant concentration (> 10⁴ CFU/mL for bronchoalveolar lavage, > 10³ CFU/mL for plugged telescoping catheter and bronchial brush). Clinical criteria for defining VAP were: a new or progressive infiltrate (without argument for cardiogenic pulmonary edema) associated with fever > 38.3°c (or leucocytes < 4000/mm^3^ or ≥ 12,000/mm^3^ and at least two clinical signs [purulent sputum, cought of dyspnoea, declining oxygenation or increased oxygen requirement or need for respiratory assistance]). From culture results, to distinguish pathogens from potential contaminants and colonizing bacteria, we reviewed the medical records (clear information on whether or not the bacterium was considered in the antibiotic therapy) and assessed whether the antibiotic therapy was effective against the bacterium. For example, if the BAL culture identified an *E. coli* and a *Coagulase-Negative Staphylococcus (CoNS)*, and the antibiotic therapy consisted of a combination of a beta-lactam and linezolid, we considered that the clinician had chosen to treat the *CoNS*.

Exclusion criteria were defined as follows: (i) Minors, (ii) Undocumented VAP cases, (iii) Non-bacterial VAP (viral, fungal), (iv) Documented VAP only by tracheal aspiration, and (v) Patients not intubated at the time of respiratory sampling.

Data were collected at each center by a local investigator or the principal investigator using an anonymized standardized paper or electronic Case Report Form (CRF). Demographic, clinical, biological, and radiological data, including past medical history, present hospital stays, and outcomes, were collected. The antibiotic exposure prior to VAP diagnosis was recorded as empirical antibiotic therapy. Definitive antibiotic therapy was defined as the final treatment based on microbiological results.

### Definition of non-targeted bacteria

Non-targeted bacteria were bacteria not included in the list of the 15 bacteria of the FAPPP. In this study, we retrospectively compared the results of bacterial cultures performed in each center to the list of these 15 bacteria. The mPCR of the FAPPP was likely not performed on most patients, so the comparison was theoretical to the list of these 15 bacteria that could have been investigated if an mPCR had been performed. Although some patients had mPCR, their results were not analyzed.

### Interpretation of the culture-positive VAP with ineffective empirical antibiotic therapy and targeted bacteria

To evaluate the potential impact of the panel results on the empirical antibiotic therapy of these culture-positive VAP (with ineffective empirical treatment), we compared the empirical antibiotic therapy with the proposed antibiotic therapy for Gram-negative bacteria. The final antibiogram provided to clinicians by the local laboratory was compared with the antibiotic therapy that would have been initiated based on ESCMID (European Society of Clinical Microbiology and Infectious Diseases) and IDSA (Infectious Diseases Society of America) recommendations, assuming the clinician had access to an mPCR result [12, 13]. This comparison was performed by one infectious disease specialist (M.R), one intensivist (K.A) and one microbiologist (E.M).

### Gram staining

Concordance between Gram staining and culture was assessed. Concordance was defined as full agreement between the Gram staining results and the final culture results. This definition was applied to both monomicrobial and polymicrobial VAP.

### Primary endpoint

The primary objective was to estimate the proportion of culture-positive VAP cases with at least one non-targeted bacterium (a bacterium that could not be detected by the FAPPP but ultimately covered by the final antibiotic therapy).

### Statistics

All statistical analyses were performed using R software (version 2024.04.2 + 764). Continuous quantitative variables were defined by their means and standard deviations. Qualitative variables were defined by their rate. For quantitative variables, Student’s t-tests or Mann-Whitney tests were used. For qualitative variables, Chi-square tests or Fisher’s exact tests were used. The false discovery rate (FDR) was used to correct for multiple comparisons. First, univariate analysis was performed to select predictor variables associated with VAP with non-targeted bacteria and then we used the Akaike Information Criterion (AIC) to decide which to include in the final model. In order to choose the most parsimonious model, a selection of variables allowing the optimization of the AIC was carried out in a backward selection process. All significant variables, as well, as the non-significant variables that interact with the other items and provide the best AIC were retained [14]. The absence of collinearity between the predictor variables was also checked using the variance inflation factor. All tests were two-tailed. A p-value threshold of 0.05 was considered significant.

The difference of culture-positive VAP with non-targeted bacteria between different ICU was assessed using Chi-square tests.

This study was authorized by the ethics committee of Angers University Hospital in March 2021 (registration number 2021-045).

## Results

### Study population

**Fig. 1:**
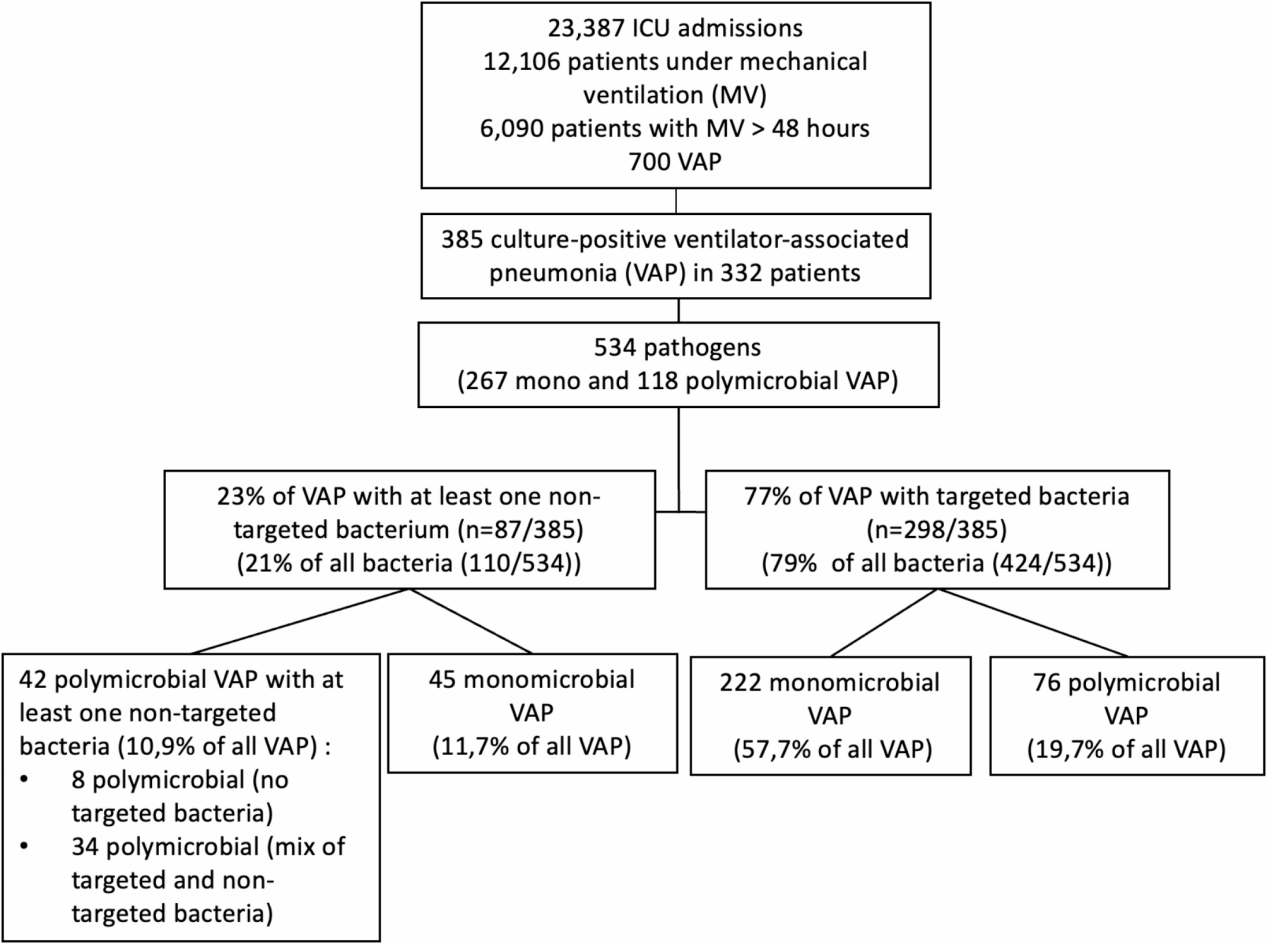
Flow chart

Three hundred and eighty-five culture-positive VAP in 332 patients from 12 ICUs were included (Fig. 1).

87% of patients experienced a single episode of culture-positive VAP, while the remaining had multiple episodes (10% had 2 VAPs, 2% had 3 VAPs and 1% had 4 VAPs). VAP was considered late-onset (> 5 days of mechanical ventilation) in 80% (*n* = 310) of cases, with a mean time of 14 ± 13 days. Antibiotic therapy had been administered for another infection prior to VAP in 84% of cases, 78% of which were initiated in the ICU. Regarding patient outcomes, 38% (*n* = 127) died in the hospital. The mean duration of mechanical ventilation was 21 ± 24 days, with an intensive care unit stay of 35 ± 38 days.

### Non-targeted bacteria

**Fig. 2:**
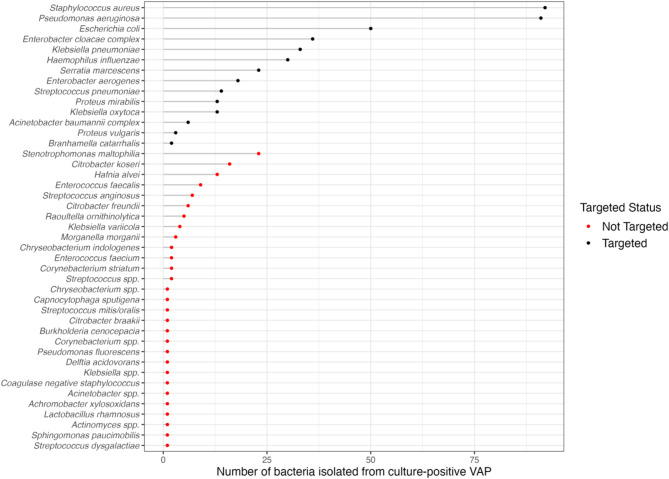
Distribution of targeted and non-targeted bacteria in culture-positive VAP

A non-targeted bacterium was observed in 23% of culture-positive VAP cases (87/385) and represented 21% (110/534) of bacteria isolated by conventional cultures and considered for antibiotic treatment by the intensivist. The distribution of targeted and non-targeted bacteria is represented in Fig. 2. Fourteen different species were considered targeted bacteria, and 29 were non-targeted. The most frequent non-targeted bacteria found in culture were *Stenotrophomonas maltophilia* (21%, *n* = 23), *Citrobacter koseri* (15%, *n* = 16), and *Hafnia alvei* (12%, *n* = 13). Polymicrobial VAP had a twofold higher risk of non-targeted bacteria (47% vs. 25%, *p* < 0.001). Gram stain was available for 93% of all culture-positive VAP episodes, with the following results: mixed morphologies (28.1%), no bacteria (27%), Gram-negative (25.5%), Gram-positive (12.2%), and unavailable (7.3%). The concordance between Gram staining performed and the Gram staining according to bacterial cultures was 42% (Supplemental results, Figure S1). For patients with multiple culture-positive VAP episodes, a first episode with a non-targeted bacteria was associated with a fourfold higher risk of subsequent culture-positive VAP with non-targeted bacteria (*p* = 0.05) (Supplemental results, Figure S2).

**Fig. 3:**
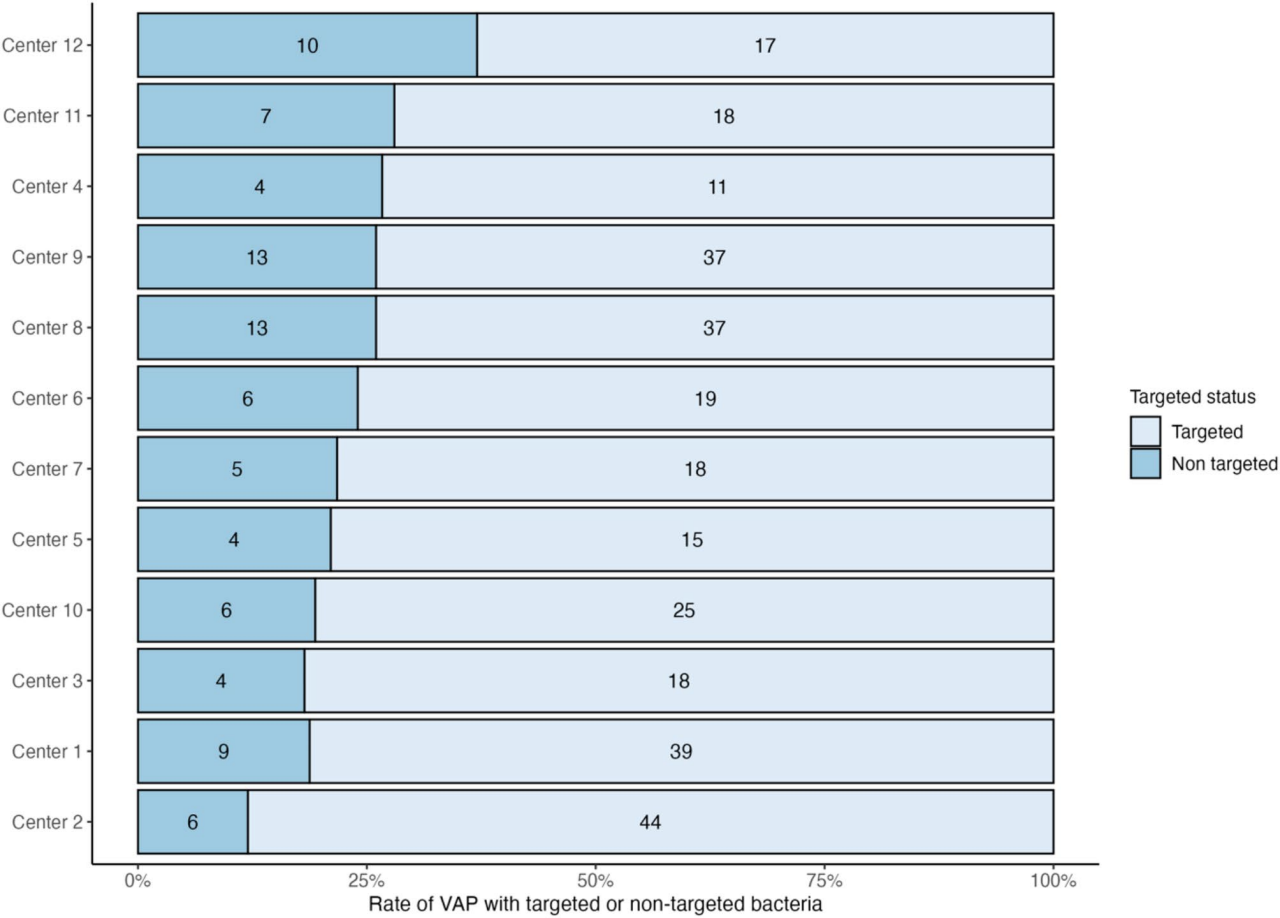
Rates of targeted and non-targeted culture-positive VAPs among the different centers. The numbers represent the count of culture-positive VAP episodes with at least one non-targeted bacteria (dark blue) or targeted bacteria (light blue)

The rate of VAP episodes with at least one non-targeted pathogen varies widely between different ICUs, ranging from 12 to 37% (Fig. 3). Using Center 2 as a reference, only Center 12 (with the highest proportion of VAP with non-targeted bacteria) has a significantly higher proportion of VAP with an OR of 4.3 (*p* = 0.013, Supplemental results, Figure S3). The bacterial species of each non-targeted bacteria by center are presented in Supplemental results, Figure S4.

### Factors associated with non-targeted bacteria

**Fig. 4:**
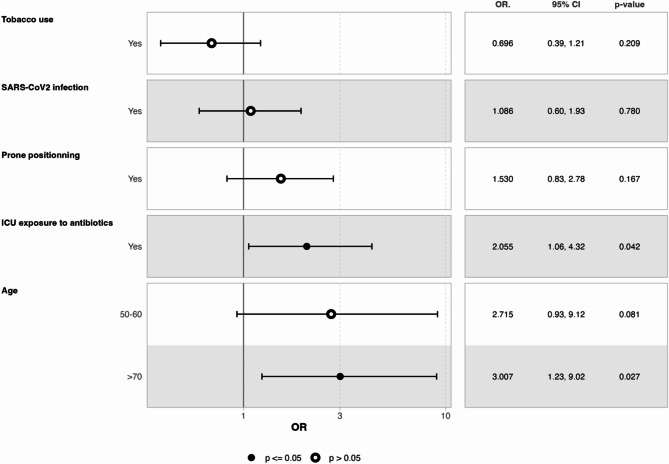
Risk factors for culture-positive VAP with non-targeted bacteria: full model of the logistic regression

The results of the univariate analysis are presented in Table 1. Age, tobacco use, in-ICU exposure to antibiotics, prone positioning, and SARS-CoV-2 infection were included in the multivariate analysis. A twofold increase in the rate of VAP with non-targeted bacteria was observed in patients with in-ICU exposure to antibiotics (25.3% versus 12.9%, *p* = 0.042) and a threefold increase for patients > 70 years old (*p* = 0.027, Fig. 4). The most parsimonious model was consistent with the full model (Supplemental results, Figure S5).

**Table 1:** Characteristics of the study population according to the panel status, univariate analysis

		Panel			
**Characteristic**	**Overall**, *N* = 385^1^	**In**, *N* = 298^1^	**Out**, *N* = 87^1^	*p-value* ^2^	**q-value** ^3^
Female sex, *n* (%)	83 (22%)	61 (20%)	22 (25%)	0.3	0.8
Age, mean (SD)	63 (13)	62 (14)	67 (12)	**0.003**	0.081
Origin of patient, *n* (%)				0.8	0.8
Home	206 (54%)	157 (53%)	49 (56%)		
Medical ward	175 (45%)	138 (46%)	37 (43%)		
Nursing home	4 (1.0%)	3 (1.0%)	1 (1.1%)		
Diabetes, *n* (%)	83 (22%)	67 (22%)	16 (18%)	0.4	0.8
Immunodepression^$^, *n* (%)	94 (24%)	75 (25%)	19 (22%)	0.5	0.8
Cirrhosis, *n* (%)	37 (9.6%)	28 (9.4%)	9 (10%)	0.8	0.8
Chronic heart disease, *n* (%)	56 (15%)	40 (13%)	16 (18%)	0.2	0.8
Chronic respiratory disease, *n* (%)	74 (19%)	61 (20%)	13 (15%)	0.2	0.8
Chronic kidney disease, *n* (%)	32 (8.3%)	25 (8.4%)	7 (8.0%)	> 0.9	> 0.9
Obesity, *n* (%)	101 (26%)	82 (28%)	19 (22%)	0.3	0.8
Tobacco use, *n* (%)	132 (34%)	109 (37%)	23 (26%)	**0.080**	0.4
Charlson score, mean (SD)	4.23 (2.83)	4.19 (2.92)	4.37 (2.50)	0.6	0.8
Knauss, *n* (%)				0.3	0.8
A	87 (23%)	73 (24%)	14 (16%)		
B	198 (51%)	146 (49%)	52 (60%)		
C	95 (25%)	75 (25%)	20 (23%)		
D	5 (1.3%)	4 (1.3%)	1 (1.1%)		
Simplified Acute Physiology Score II, mean (SD)	50 (17)	50 (17)	51 (18)	0.8	0.8
Unknown	3	3	0		
Sequential Organ Failure Assessment score, mean (SD)	7.5 (3.9)	7.5 (3.9)	7.6 (3.9)	0.8	0.8
Reason for admission, *n* (%)				0.6	0.8
Emergency surgery	28 (7.3%)	24 (8.1%)	4 (4.6%)		
Medical	350 (91%)	268 (90%)	82 (94%)		
Scheduled surgery	7 (1.8%)	6 (2.0%)	1 (1.1%)		
Ventilator-associated pneumonia (VAP) number, *n* (%)				0.5	0.8
1	332 (86%)	258	74		
2	41 (11%)	29	12		
3	9 (2.3%)	8	1		
4	3 (0.8%)	3	0		
Delay before VAP, mean (SD)	14 (13)	13 (13)	15 (14)	0.4	0.8
Antibiotic before ICU, *n* (%)	140 (36%)	107 (36%)	33 (38%)	0.7	0.8
In-ICU antibiotic exposure, *n* (%)	300 (78%)	224 (75%)	76 (87%)	**0.016**	0.2
Acute respiratory distress syndrome, *n* (%)				0.3	0.8
Mild ARDS	93 (24%)	74 (25%)	19 (22%)		
Moderate ARDS	196 (51%)	150 (50%)	46 (53%)		
No ARDS	23 (6.0%)	21 (7.0%)	2 (2.3%)		
Severe ARDS	73 (19%)	53 (18%)	20 (23%)		
Prone positioning, *n* (%)	79 (21%)	55 (18%)	24 (28%)	**0.064**	0.4
Extracorporeal membrane oxygenation, *n* (%)	10 (2.6%)	7 (2.3%)	3 (3.4%)	0.7	0.8
Vasopressor, *n* (%)	162 (42%)	123 (41%)	39 (45%)	0.6	0.8
Renal replacement therapy, *n* (%)	52 (14%)	42 (14%)	10 (11%)	0.5	0.8
SARS-CoV2 infection, *n* (%)	109 (28%)	78 (26%)	31 (36%)	0.085	0.4

^1^*n* (%); Mean (SD)

^2^Pearson’s Chi-squared test; Welch Two Sample t-test; Fisher’s exact test

^3^False discovery rate correction for multiple testing

^$^Defined by any immunosuppressive agent, active cancer, graft

The distribution of the age of patients with culture-positive VAP episodes, including those with targeted and non-targeted bacteria, is presented in Supplemental results, Figure S6, and the proportion of in-ICU exposure to antibiotics is shown in Supplemental results, Figure S7.

### Empiric and definitive antibiotic therapies

**Fig. 5:**
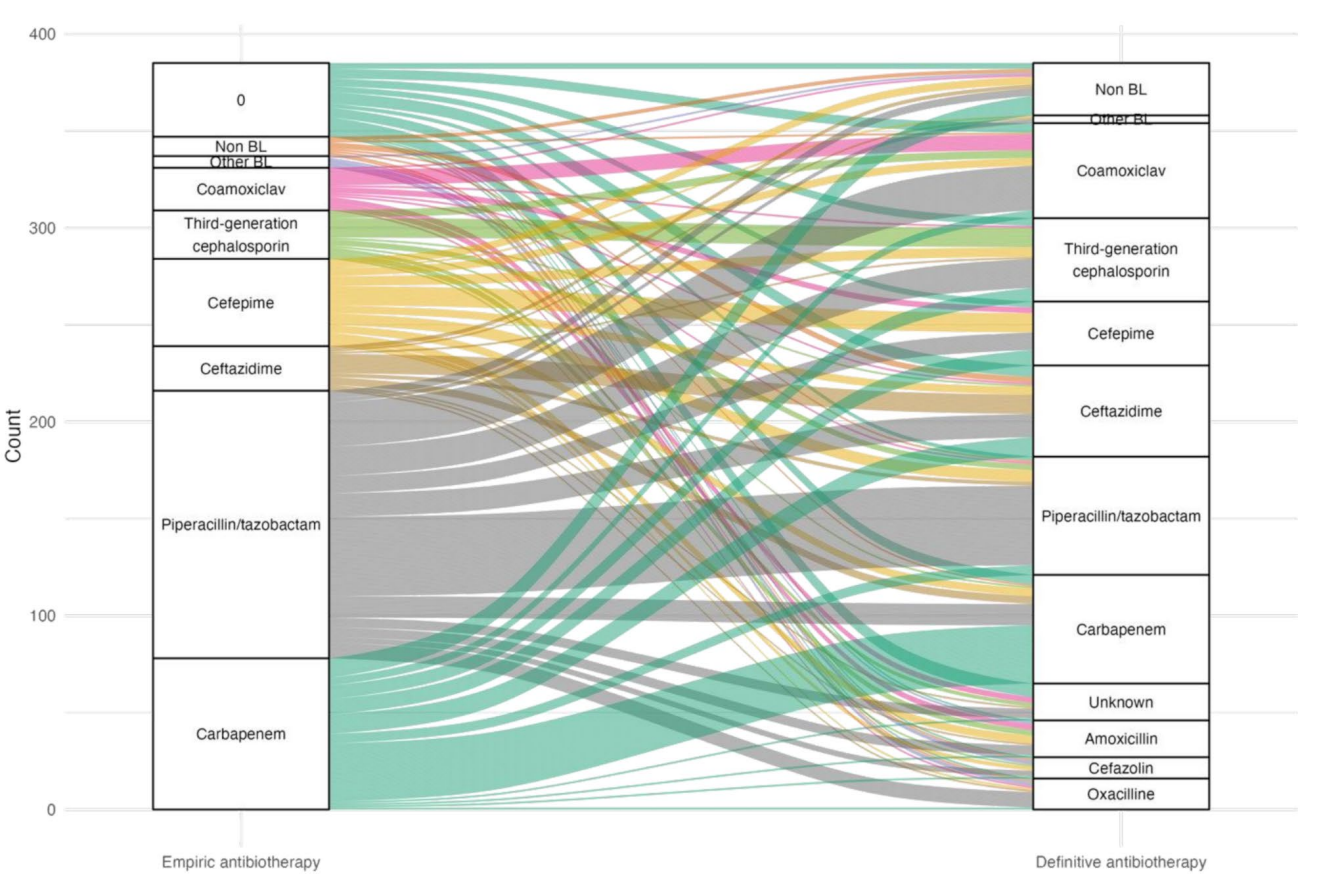
Sankey diagram of empiric and definitive antibiotic therapies

77.9% of culture-positive VAP episodes had prior exposure to antibiotics, mainly third-generation cephalosporins (3GC) (49%, *n* = 150), co-amoxiclav (15%, *n* = 57), and piperacillin/tazobactam (13%, *n* = 49). 90.1% (*n* = 347) of culture-positive VAP episodes received empiric antibiotic therapy. Empiric and definitive antibiotic therapies for each culture-positive VAP episode are represented in the Sankey diagram (Fig. 5). Piperacillin/tazobactam (35.8%, *n* = 138/385), carbapenems (20.3%), and cefepime (11.7%) were the most frequent empiric antibiotic therapies, whereas piperacillin/tazobactam (15.8%), carbapenems (14.5%), and co-amoxiclav (12.7%) were the most frequent for definitive antibiotic therapies. After exclusion of the 37 episodes with no information on empirical antibiotics, 86.2% of culture-positive VAP episodes received effective antibiotics.

21 culture-positive VAP cases actually underwent a FAPPP. However, the sample size was too small to determine whether the technique had an impact on these episodes.

### Culture-positive VAP with ineffective antibiotic therapy

For the 48 culture-positive VAP cases (12.5%) with ineffective empiric treatment, 76 bacteria were identified by culture. The five most common bacteria were *Pseudomonas aeruginosa* (*n* = 17), *Stenotrophomonas maltophilia* (*n* = 11), *Enterobacter cloacae complex* (*n* = 6), *Staphylococcus aureus* (*n* = 6), *Citrobacter koseri* (*n* = 5), *Escherichia coli* (*n* = 5), and *Klebsiella pneumoniae* (*n* = 5). 56% (*n* = 27) of these VAP cases had targeted bacteria. The five most common bacteria for these VAP cases were *Pseudomonas aeruginosa* (*n* = 11), *Enterobacter cloacae complex* (*n* = 6), *Klebsiella pneumoniae* (*n* = 4), *Staphylococcus aureus* (*n* = 4), and *Enterobacter aerogenes* (*n* = 3). 67% of these VAP cases with ineffective empiric treatment (18/27) and targeted bacteria could have had effective empirical antibiotic therapy if the panel result had been available. On the other hand, the panel result probably would not have changed anything for the other 9 VAP cases (Supplemental results, Table S2).

## Discussion

In this study conducted in 12 ICUs in a large region of France, we were able to identify that at least one non-targeted bacterial species was involved in 23% of culture-positive VAP (range 12–37%) cases over the course of 385 culture-positive VAP episodes. *S. maltophilia* was one of the main non-targeted pathogens by the FAPPP. Several Enterobacteriaceae not targeted by the panel, such as *H. alvei* and *C. koseri*, were also frequently observed with variable distributions across centers.

Dessajan et al. [15], found a proportion of non-targeted bacteria of 9% (from 0 to 31%) among 15 pooled studies, of which 8 were performed in ICUs [16–23]. A recent meta-analysis of 30 studies, including 21 in ICUs [16–36], involving nearly 9000 samples including 4000 BAL, demonstrated that 9.3% [95% CI 9.2–9.5] of strains identified in culture were outside the panel [37]. Twenty-five studies reported a total of 579 positive culture strains outside the FAPPP (mean number of non-targeted bacteria of 9.3%), mainly *Stenotrophomonas maltophilia* (24% of cases), followed by *Citrobacter koseri* (10% of cases) and *Morganella morganii* (6%). *Hafnia alvei*,* Providencia spp.*,* Raoultella spp.*, and numerous non-fermenting GNB were also isolated [38]. Few studies focused on VAP despite a specific microbiology and guidelines for empirical antibiotic therapy [39]. The proportion of antibiotic modifications seems however to be high in VAP in comparison with health-care associated pneumonia or community-acquired pneumonia [40]. Ultimately, it is likely that the proper use of the panel requires knowledge of the local epidemiology to account for any non-targeted bacteria. A recent study by Ibn Saied et al., based on the multicentre OUTCOMEREA database, indicated that over 6% of VAP cases were related to *Stenotrophomonas maltophilia* [41]. The significant proportion of VAP cases involving a “non-targeted bacteria” in our study complicates the interpretation of a FAPPP test, especially in patients over 70 or those exposed to antibiotics in the ICU.

While the causes of ineffective empirical antibiotic therapies are numerous, we can list the main causes of failure: not accounting for a third-generation cephalosporin resistant (3GCR) Enterobacteriaceae, not using an anti-pseudomonal beta-lactam, the presence of a multidrug resistant *P. aeruginosa*, the presence of methicillin resistant *S. aureus* (MRSA), the presence of *S. maltophilia*, etc. By detecting CTX-M, MRSA, and *P. aeruginosa*, the FAPPP can theoretically address a large proportion of these gaps in empirical antibiotic therapy. In a simulation of the potential impact of the mPCR, a French team estimated that among 85 ICUs patients (75% with VAP), the use of a mPCR would have resulted in only one ineffective empirical antibiotic therapy [15]. In our study, we found that 12.5% of empirical therapies were ineffective. Additionally, for 59% of the culture-positive VAP cases with ineffective empiric treatment and targeted bacteria (16/27), the use of the FAPPP could have led to effective empirical antibiotic therapy according to our simulation. Therefore, while the simulated impact observed in our study is encouraging, it requires validation through randomized controlled trials.

While the majority of VAP cases receive effective empirical antibiotic therapy, it is far from optimal, with an overly broad use of piperacillin-tazobactam or carbapenems (35.8% and 20.3% respectively in our study). The results of an mPCR test like the FAPPP could enable early optimization of antibiotic therapy. In the previously mentioned study by the French team, the implementation of mPCR resulted in adjustments to antibiotic treatments in 66% of pneumonia episodes (63 out of 95). Specifically, early initiation of effective antibiotics occurred in 21% of patients (20 out of 95), while early de-escalation was achieved in 39% of patients (37 out of 95) [15, 42]. Similar results were observed with other teams [16, 43]. In a real-life studies, Maataoui et al. showed that a negative FAPPP test avoided 28% of antibiotic therapy in VAP patients during the COVID-19 epidemic [21].

Some teams have proposed a probabilistic antibiotic therapy algorithm based on both Gram and early FAPPP results, with re-evaluation in all cases at 48 h with culture results. The clinical results of such an approach remain to be determined [44]. Given the performance of Gram staining in VAP, we question an approach that incorporates its results (42% concordance in our study). A large recent studies on 206 VAP found that Gram-stain was concordant with culture in 74% of episodes [45]. However, some of the discrepancies may have been due to Gram stain sensitivity, which can be influenced by the bacterial inoculum in VAP and potentially affected by prior antibiotic therapy.

Globally, mPCR like the FAPPP and Unyvero Pneumonia Panel (Curetis), performed similarly and have been considered more sensitive than routine microbiology, detecting potential pathogens in patient samples reported as culture negative [26]. The enhanced detection sensitivity achieved through PCR holds promise for more effective and targeted antimicrobial prescribing. However, the combination of positive FAPPP and negative culture could mean the presence of bacteria at low inoculums, which could be not considered for treatment or could refer to the presence of bacterial DNA corresponding to resolved infections or an active antibiotic treatment at the time of sampling. Nevertheless, rapid advancements in diagnostic techniques are expected in the near future, including the increasing use of metagenomics (Next-Generation Sequencing), which enables broad-spectrum amplification of pathogen DNA [46–49]. This will once again challenge our understanding of gold standard tests and the complex task of distinguishing colonization from infection, as well as active infection from resolved infection in VAP. The cost of mPCR techniques raises questions about their routine use. In our study, only 5% (18/385) of VAP cases appeared to benefit from mPCR to improve the rate of effective empiric antibiotic therapy. However, the combined benefits of these tools (non-prescription, early adaptation of antibiotic therapy) are greater and warrant further study.

The strengths of this study include its multicenter design, which allowed for the inclusion of numerous patients, and its relatively recent study period. Additionally, only high-quality specimens were tested, providing a robust reflection of VAP epidemiology. The study’s limitations are inherent to its retrospective nature, including missing or lost data, and the fact that the epidemiology of this region may differ from that of other parts of the world. Another limitation lies in the method used to classify bacteria as pathogens or colonizers. Some clinicians may have mistakenly decided to treat a bacterium that was not responsible for the VAP but was found in the culture. In such situations, which are nonetheless a source of bias, we did not reinterpret the clinicians’ decisions. By excluding tracheal aspirations, we also limited the number of VAP cases included in the study, which could restrict its generalizability. Finally, culture-negative VAP cases were not included in this study, although their frequency in the ICU might not be negligible (data not collected).

## Conclusion

With 23% of culture-positive VAP cases having at least one non-targeted bacterium identified by the FAPPP, this mPCR is not sufficiently reliable to exclude a bacterial infection. However, the FAPPP could allow for the early identification of bacterial species not considered in standard empirical antibiotic therapy, enabling the proposal of personalized empirical therapy. An updated version of the panel should incorporate the main currently non-targeted bacteria to further optimize its performance.

## Electronic supplementary material

Below is the link to the electronic supplementary material.

Supplementary Material 1: **Figure S1**. Concordance between Gram staining performed and the Gram staining according to bacterial cultures. **Figure S2**. Sankey diagram of targeted and non-targeted VAP for patients with multiples episodes of VAP. **Figure S3**. Odds ratio for the risk of VAP with non-targeted bacteria in each center, using Center 2 as the reference. **Figure S4**. Bacterial species of each non-targeted bacteria by center. **Figure S5**. Restricted model of the logistic regression for factors associated with VAP episodes involving non-targeted bacteria. **Figure S6**. Distribution of the age of patients with VAP episodes, according to the status of the VAP episode. Vertical dashed lines represent the mean age of patients by category. **Figure S7**. Proportion of in-ICU antibiotic exposure for patients with VAP episodes involving targeted and non-targeted bacteria.

## Data Availability

The datasets used and/or analysed during the current study are available from the corresponding author on reasonable request.

## Abbreviations

3GC: Third Generation Cephalosporin
AIC: Akaike Information Criterion
ARDS: Acute Respiratory Distress Syndrome
BL: Beta Lactam
BAL: Bronchoalveolar Lavage
CI: Confidence Interval
COVID-19: Coronavirus Infectious Disease 2019
CRF: Case Report Form
DNA: Deoxyribonucleic acid
ESCMID: European Society of Clinical Microbiology and Infectious Diseases
FAPPP: FilmArray^®^ Pneumonia Panel Plus^®^
FDR: False Discovery Rate
ICU: Intensive Care Unit
IDSA: Infectious Diseases Society of America
IQR: Interquartile Range
MDR: Multidrug-Resistant
MV: Mechanical Ventilation
NGS: Next-Generation Sequencing
OR: Odds Ratio
mPCR: multiplex Polymerase Chain Reaction
SARS-CoV2: Severe Acute Respiratory Syndrome Coronavirus 2
SD: Standard Deviation
VAP: Ventilator-Associated Pneumonia
