# Supplementary material for: Epidemiology and prediction of non-targeted bacteria by the filmarray pneumonia plus panel in culture-positive ventilator-associated pneumonia: a retrospective multicentre analysis
Source: Ann Intensive Care. 2025 Apr 28;15:57. doi: 10.1186/s13613-025-01468-6 (PMC12037957; doi:10.1186/s13613-025-01468-6)
Supplement: Supplementary file 1 — Supplementary Material 1: Figure S1. Concordance between Gram staining performed and the Gram staining according to bacterial cultures. Figure S2. Sankey diagram of targeted and non-targeted VAP for patients with multiples episodes of VAP. Figure S3. Odds ratio for the risk of VAP with non-targeted bacteria in each center, using Center 2 as the reference. Figure S4. Bacterial species of each non-targeted bacteria by center. Figure S5. Restricted model of the logistic regression for factors associated with VAP episodes involving non-targeted bacteria. Figure S6. Distribution of the age of patients with VAP episodes, according to the status of the VAP episode. Vertical dashed lines represent the mean age of patients by category. Figure S7. Proportion of in-ICU antibiotic exposure for patients with VAP episodes involving targeted and non-targeted bacteria. [file 13613_2025_1468_MOESM1_ESM.docx]

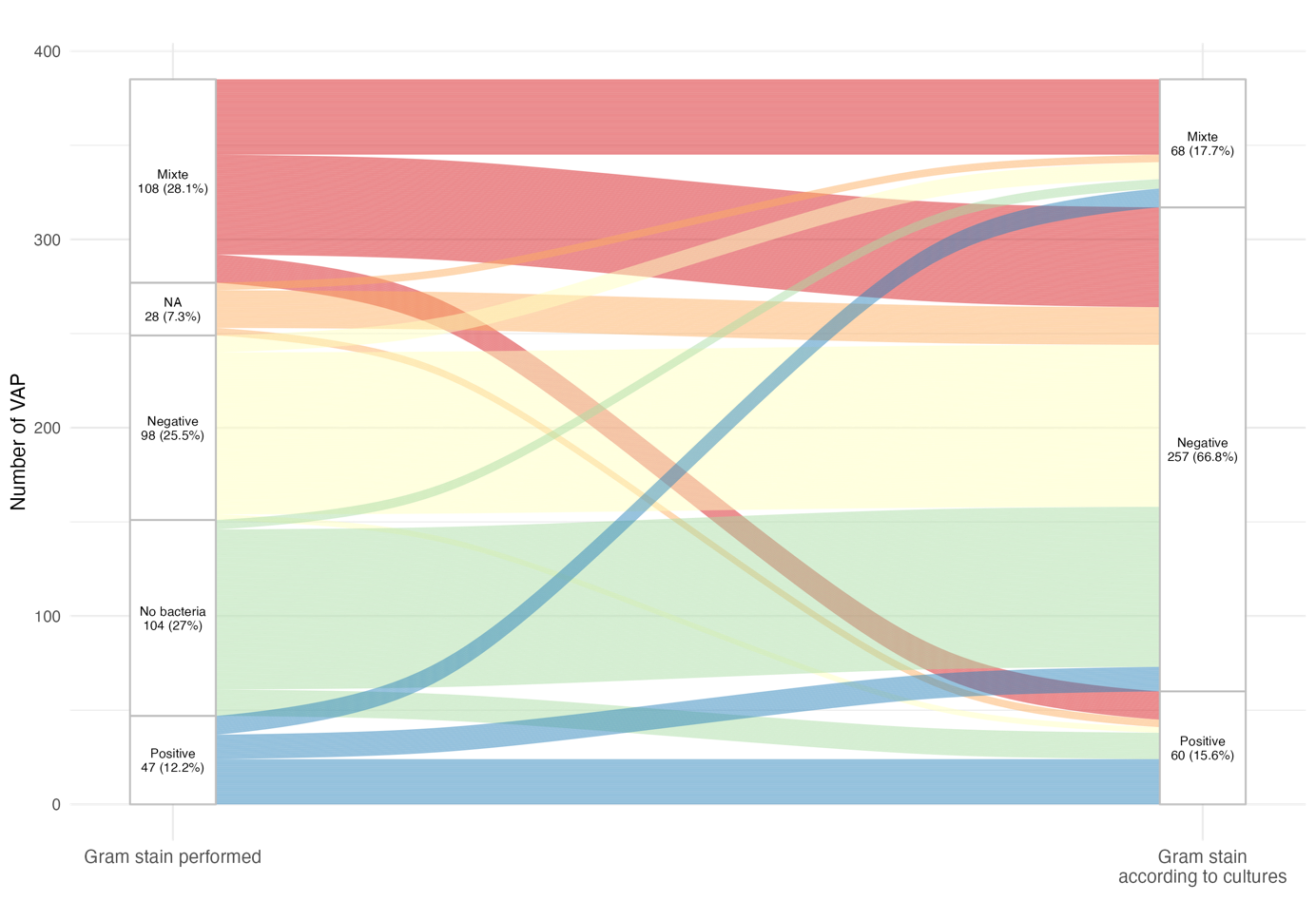


**Figure S1**: Concordance between Gram staining performed and the Gram staining according to bacterial cultures.


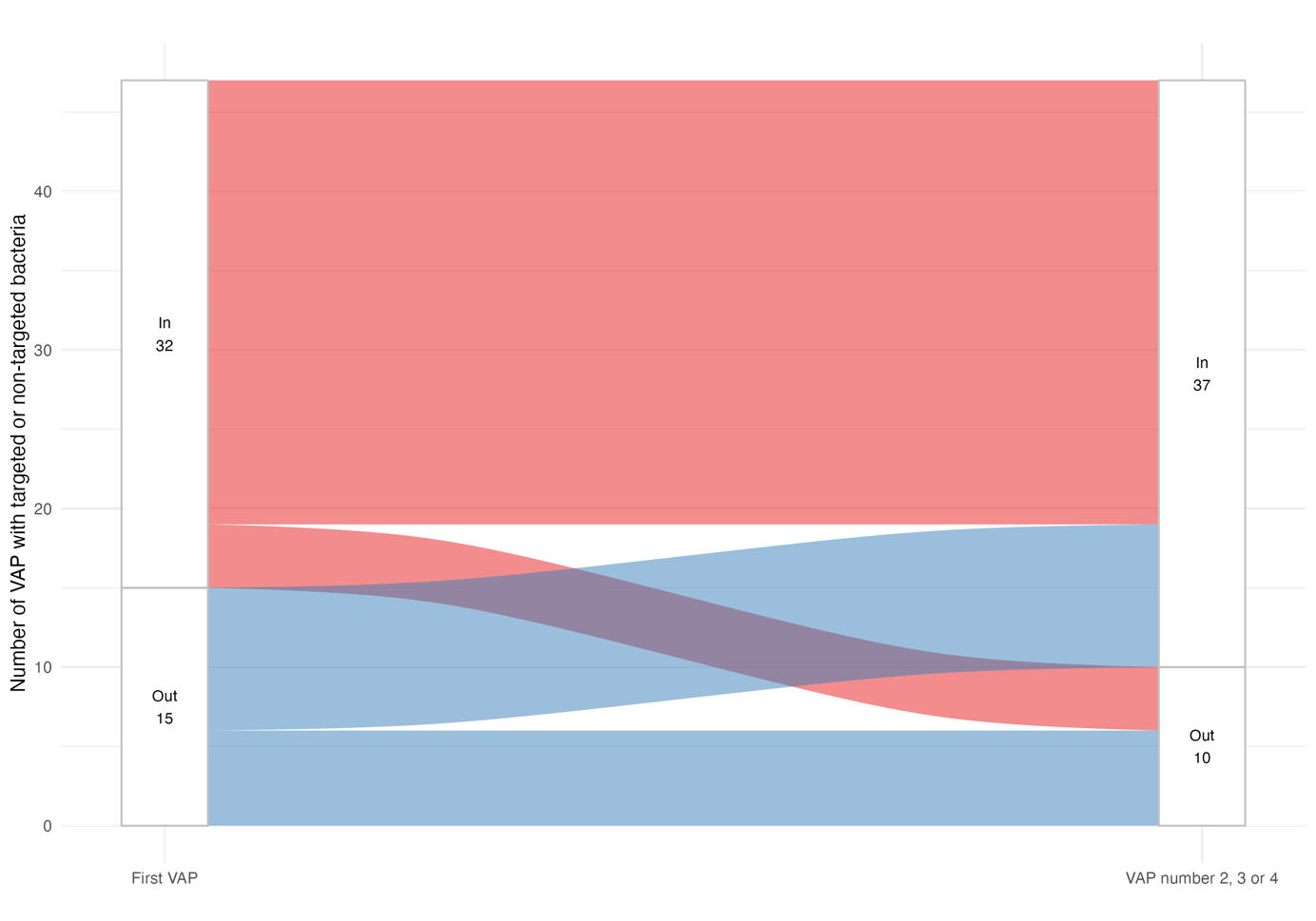


**Figure S2**: Sankey diagram of targeted and non-targeted VAP for patients with multiples episodes of VAP.


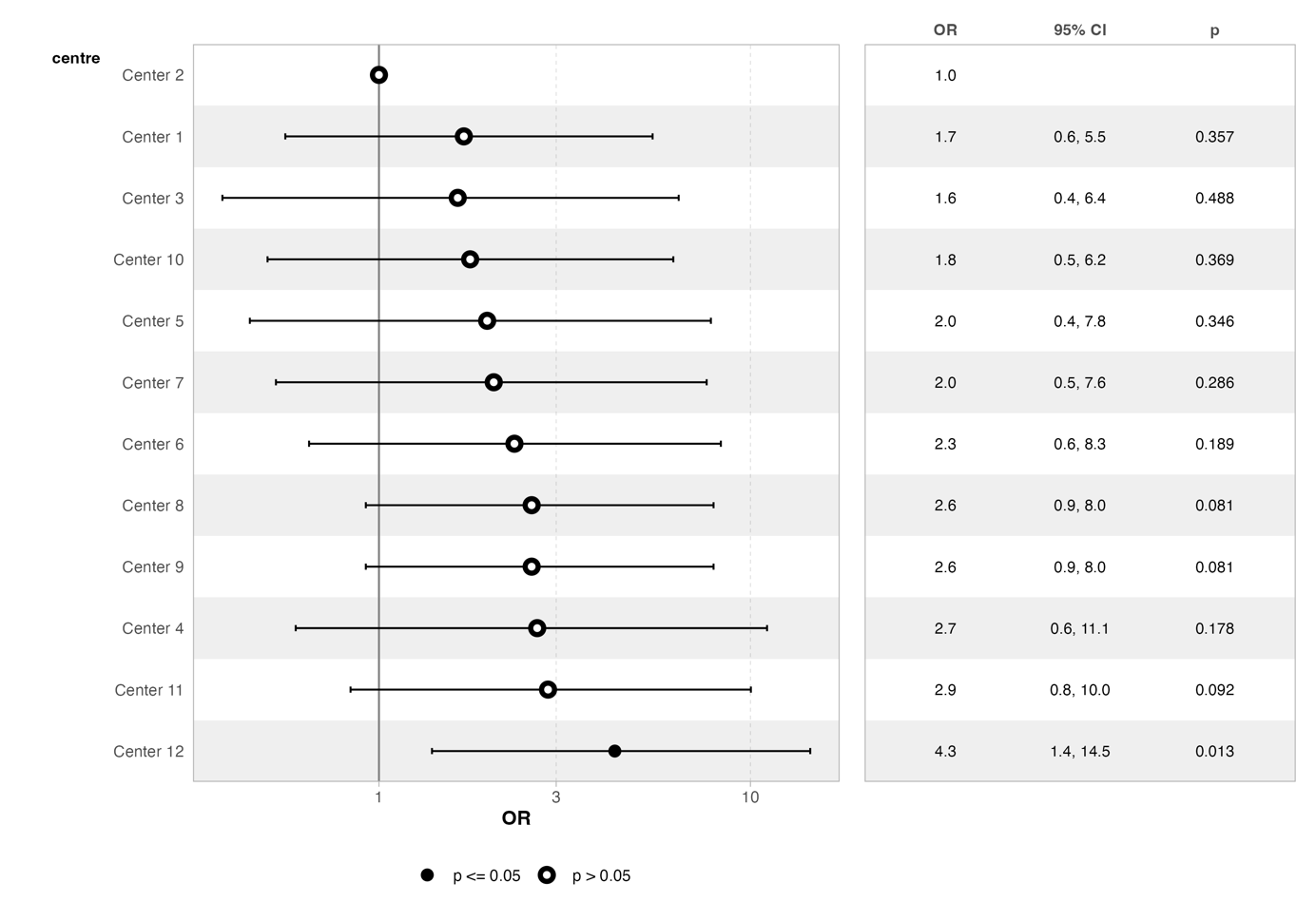


**Figure S3**: Odds ratio for the risk of VAP with non-targeted bacteria in each center, using Center 2 as the reference.​


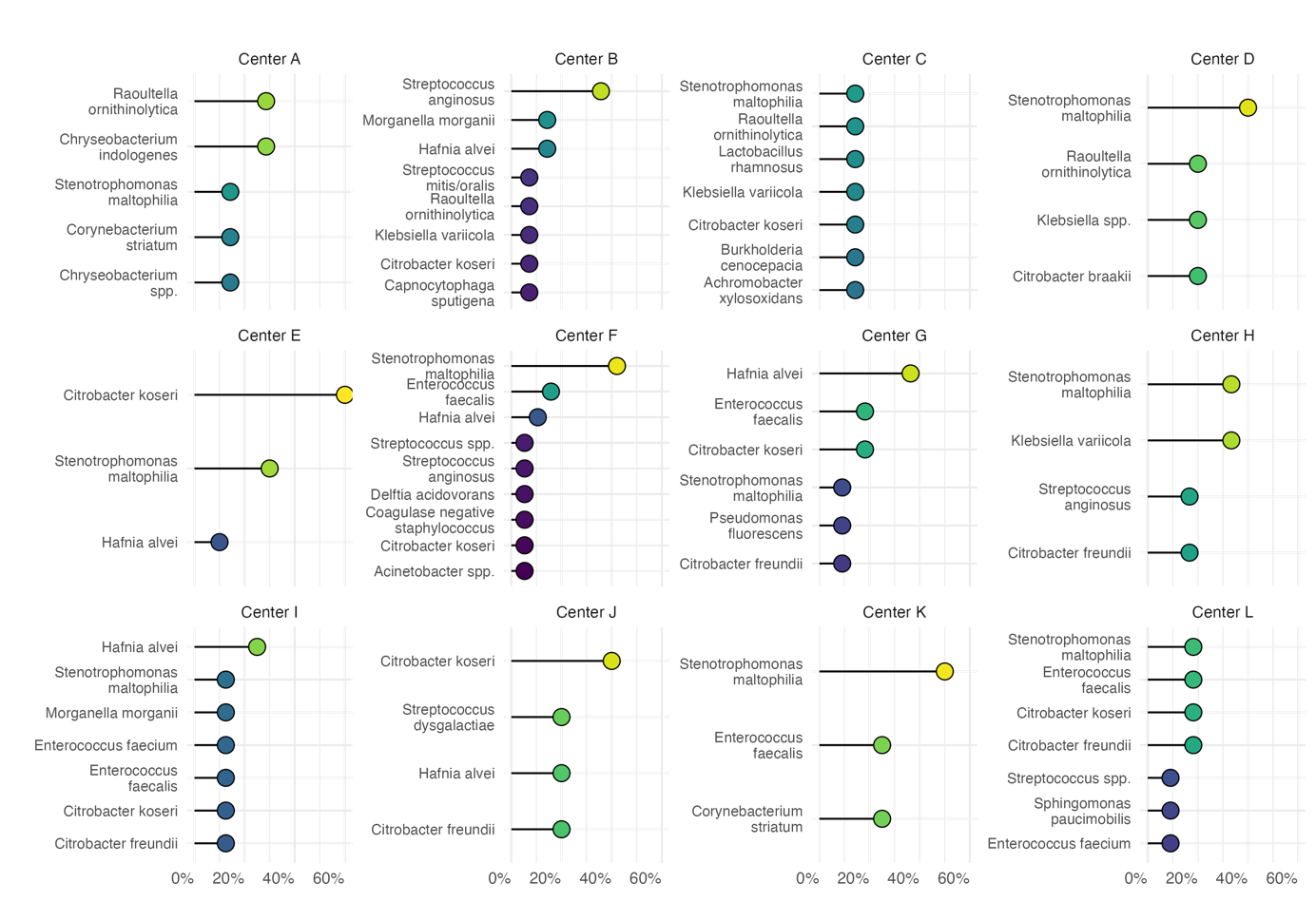


**Figure S4**: Bacterial species of each non-targeted bacteria by center


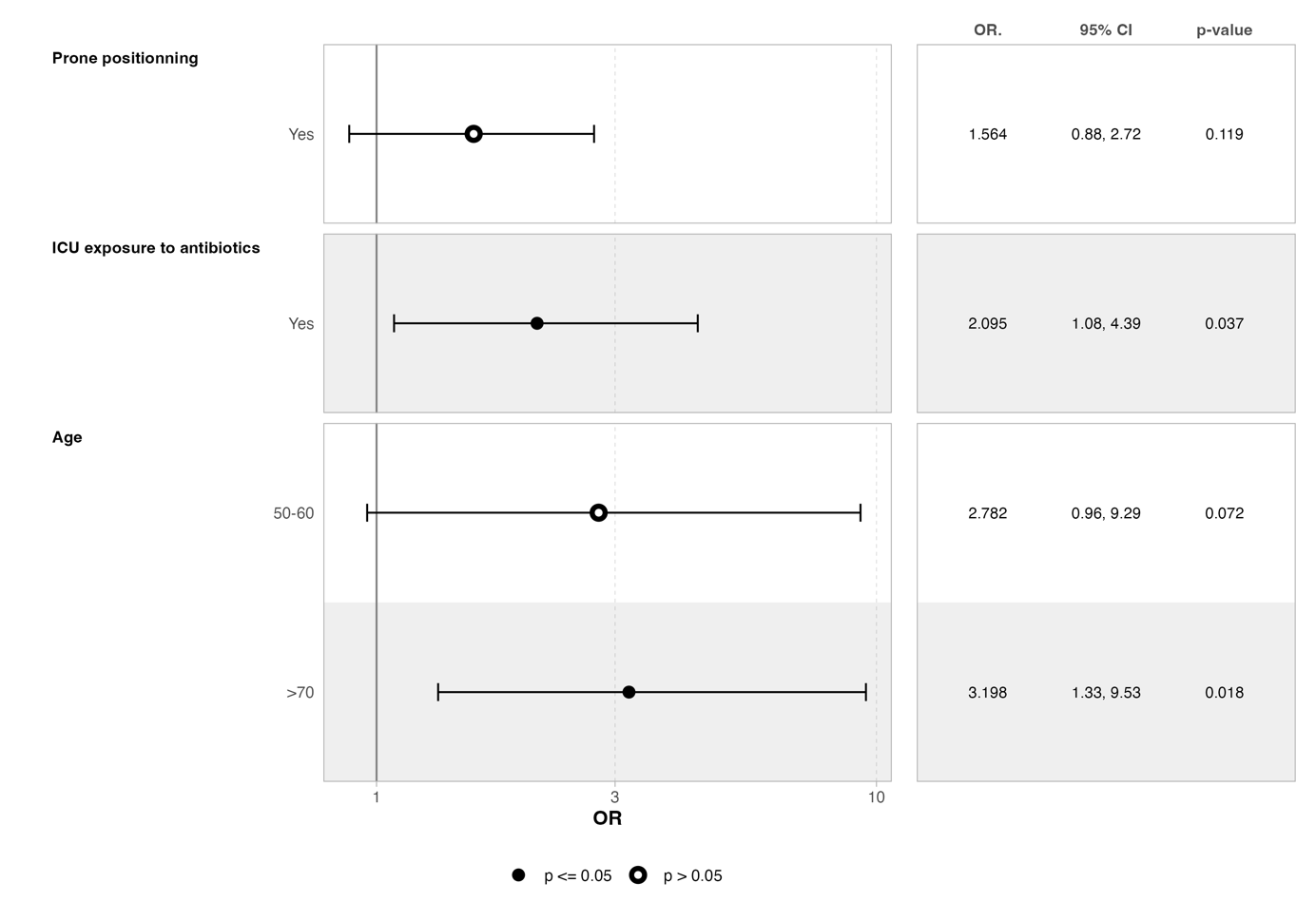


**Figure S5**: Restricted model of the logistic regression for factors associated with VAP episodes involving non-targeted bacteria.


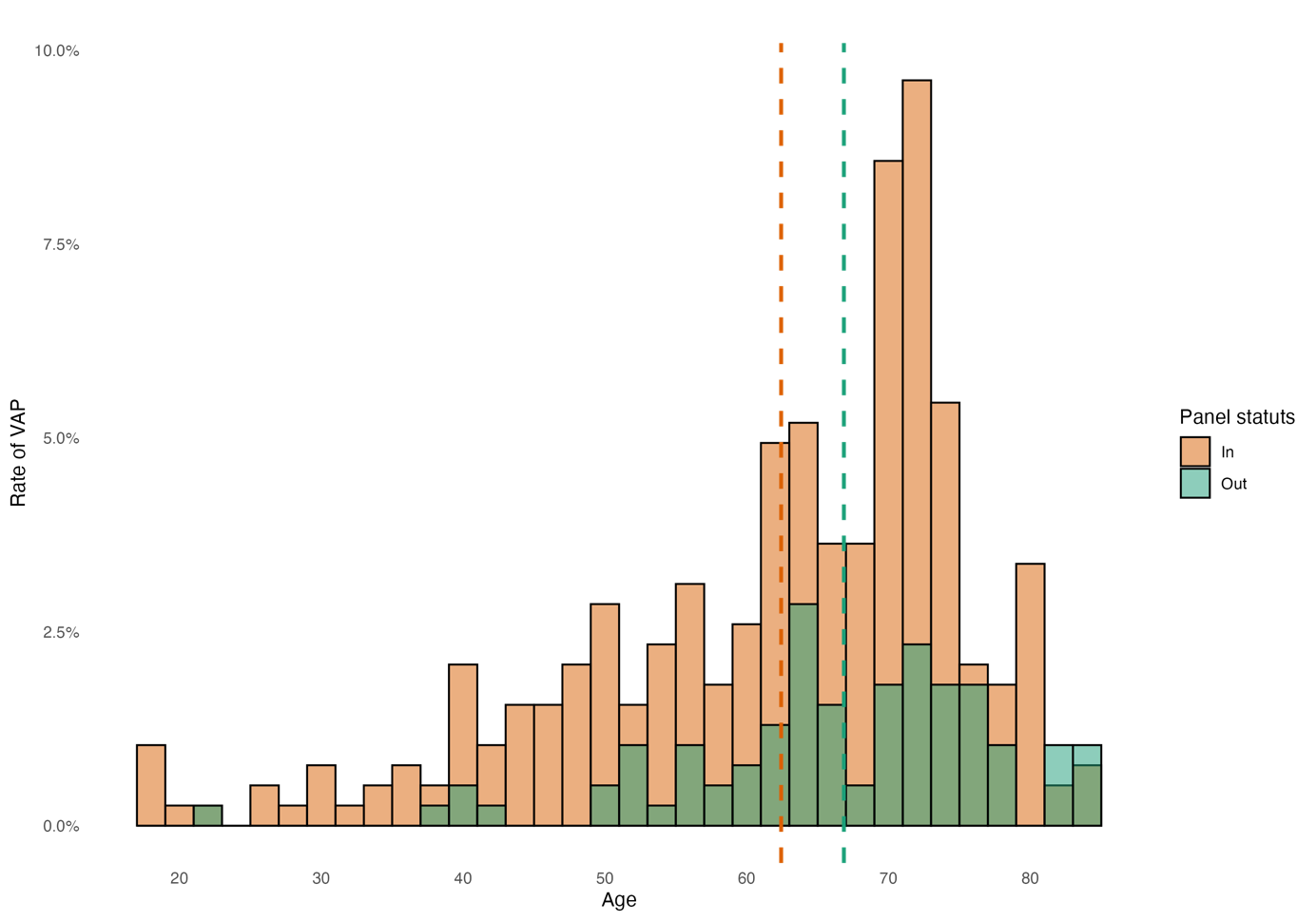


**Figure S6**: Distribution of the age of patients with VAP episodes, according to the status of the VAP episode. Vertical dashed lines represent the mean age of patients by category.


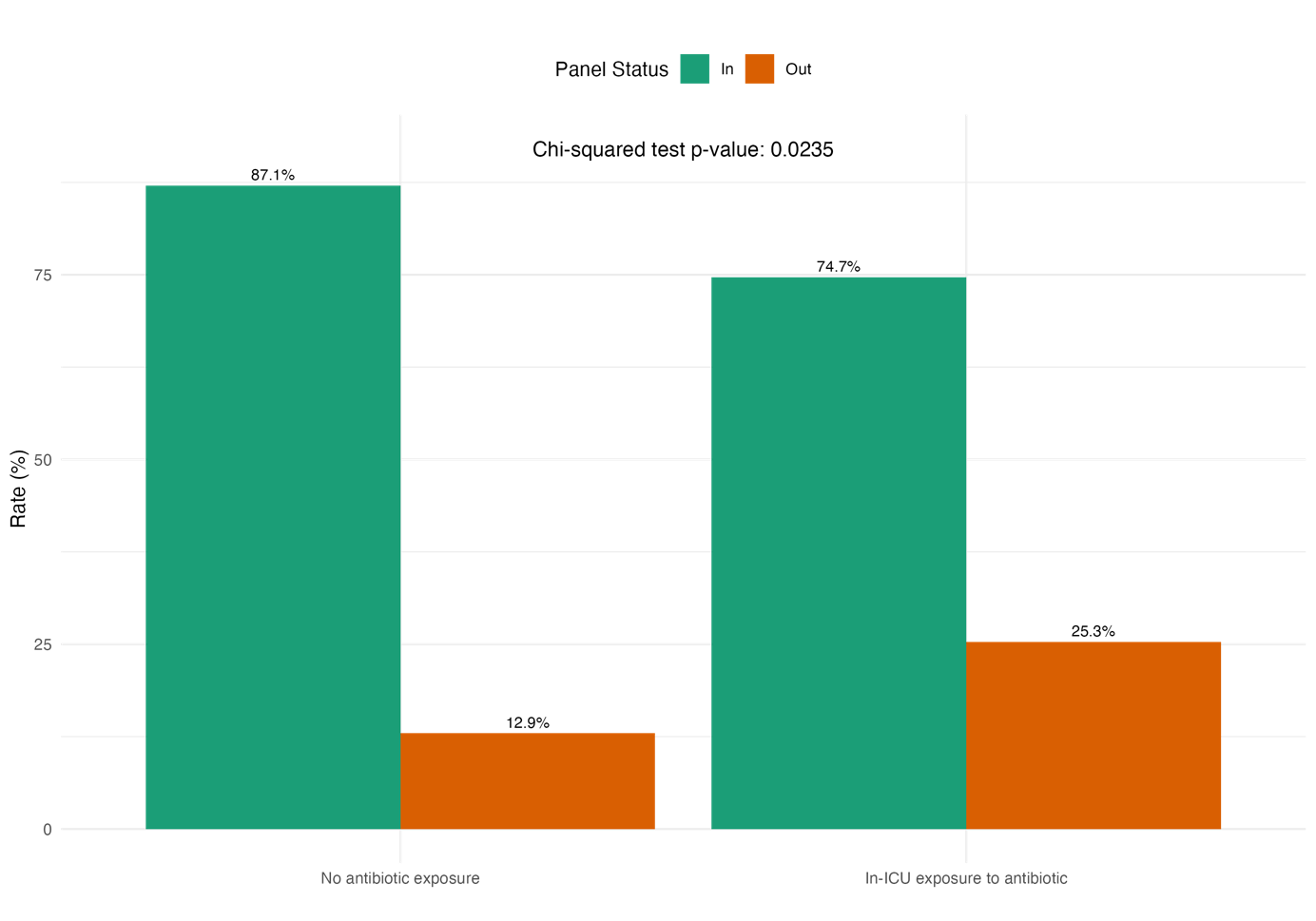


**Figure S7**: Proportion of in-ICU antibiotic exposure for patients with VAP episodes involving targeted and non-targeted bacteria.​

| **Table S2: Ventilator-associated pneumonia with ineffective empirical antibiotic therapy and targeted bacteria** | | | | | | | | | |
| --- | --- | --- | --- | --- | --- | --- | --- | --- | --- |
| **VAP no.** | **Bacterial species no. 1** | **Commentary** | **Bacterial species no. 2** | **Commentary** | **Empirical antibiotic therapy** | **Reason n.1 for ineffective therapy** | **Proposition of empirical therapy theoretically based on panel result** | **Potential impact of FilmArray® Pneumonia Panel plus** | **Commentary** |
| 1 | P. aeruginosa | Wild-type for beta-lactam |  |  | Coamoxiclav | Absence of an APBL | APNCBL |  |  |
| 2 | P. aeruginosa | APNCBL resistant |  |  | Ceftazidime | APNCBL resistant | APNCBL |  | APNCBL would not have been effective |
| 3 | P. mirabilis | WT | E. coli | ESBL | Pip/taz | ESBL R to pip/taz | Carbapenem |  |  |
| 4 | P. aeruginosa | R to pip/taz carbapenem. S to ceftza |  |  | Pip/taz |  | APNCBL |  | Pip/taz R, cefta S |
| 5 | S. pneumoniae |  | K. pneumoniae | ESBL | Pip/taz | ESBL R to pip/taz | Carbapenem |  | CTX-M are included in the panel |
| 6 | P. aeruginosa | Wild-type for BL | E. cloacae cx | dAmpC | Ceftazidime | dAmpC | Cefepime or pip/taz |  | Cefepime and pip/taz ineffective on the E. cloacae cx |
| 7 | P. aeruginosa | APNCBL resistant |  |  | Ceftazidime | Ceftazidime R | APNCBL |  | APNCBL would not have been effective |
| 8 | E. cloacae cx | dAmpC; cefepime S | P. aeruginosa | WT for BL | Ceftazidime | Ceftazidime R | Cefepime |  | Cefepime was considered effective |
| 9 | E. aerogenes | dAmpcC; cefepime S |  |  | Pip/taz | Pip/taz R | Cefepime |  | Cefepime was considered effective |
| 10 | E. coli | TEM resistant inhibitor |  |  | Pip/taz | Pip/taz R | 3GC |  | 3GC S |
| 11 | P. aeruginosa | WT for BL | S. pneumoniae |  | Ceftazidime | S. pneumoniae R to cefta | Pip/taz |  |  |
| 12 | P. aeruginosa | APNCBL resistant |  |  | Ceftazidime | Ceftazidime R | APNCBL |  | APNCBL would not have been effective |
| 13 | E. faecalis | WT | K. pneumoniae | TEM | Cefepime | E. faecalis R | Pip/taz |  | K. pneumoniae R to pip/taz |
| 14 | P. aeruginosa | WT for BL | H. influenzae |  | Ceftriaxone |  | Pip/taz |  | H. influenzae S to pip/taz |
| 15 | E. cloacae cx. | ESBL |  |  | Cefepime |  | Carbapenem |  | CTX-M are included in the panel |
| 16 | E. cloacae cx. | dAmpC |  |  | Pip/taz | dAmpC | Cefepime |  | Cefepime R |
| 17 | P. aeruginosa | R to BL except Pip/taz | K. pneumoniae | ESBL | Linezolid |  | Carbapenem |  | P. aeruginosa R to carbapenem |
| 18 | P. aeruginosa | WT for BL | S. aureus | MRSA | Carbapenem | MRSA | Ceftazidime + linezolid |  | MRSA is included in the panel |
| 19 | E. cloacae cx. | dAmpC |  |  | Cefepime | Cefepime R | Cefepime |  | dAmpC is not detected; Cefepime R |
| 20 | H. influenzae | Coamoxiclav R |  |  | Pip/taz | Pip/taz R | 3GC |  | 3GC S |
| 21 | E. cloacae cx. | ESBL |  |  | Cefepime |  | Carbapenem |  | CTX-M are included in the panel |
| 22 | P. mirabilis | Penicillinase | S. aureus | MRSA | 3GC |  | 3GC + linezolid |  | MRSA is included in the panel |
| 23 | E. coli | TEM resistant inhibitor |  | TEM | Pip/taz |  | 3GC |  | 3GC S |
| 24 | S. aureus | MSSA | E. aerogenes | WT for BL | Coamoxiclav | AmpC | Cefepime + cefazolin |  | MSSA is included in the panel |
| 25 | E. aerogenes | dAmpC; cefepime S |  |  | Pip/taz | dAmpC | Cefepime |  | Cefepime was considered effective |
| 26 | K. pneumoniae | ESBL |  |  | 3GC | ESBL | Carbapenem |  | CTX-M are included in the panel |
| 27 | S. aureus | MSSA | K. oxytoca | WT for BL | Linezolid | Enterobacteriaceae | Pip/taz +/- linezolid |  | WT for both |
| 3GC: Third-generation cephalosporin; APBL: Anti-pseudomonal beta-lactam; APNCBL: Anti-pseudomonal non-carbapenem beta-lactam resistant; BL: Beta-lactam; Pip/taz: Piperacillin/tazobactam; WT: Wild-type | | | | | | | | | |
| Novel BL with an antipseudomonal activity (ceftazidime-avibactam; ceftolozane-tazobactam) are not considered in this table. | | | | | | | | | |
